# Supplementary figures and images for: The CXCR6-CXCL16 axis mediates T cell control of polyomavirus infection in the kidney
Source: PLoS Pathog. 2025 Mar 5;21(3):e1012969. doi: 10.1371/journal.ppat.1012969 (PMC11922244; doi:10.1371/journal.ppat.1012969)

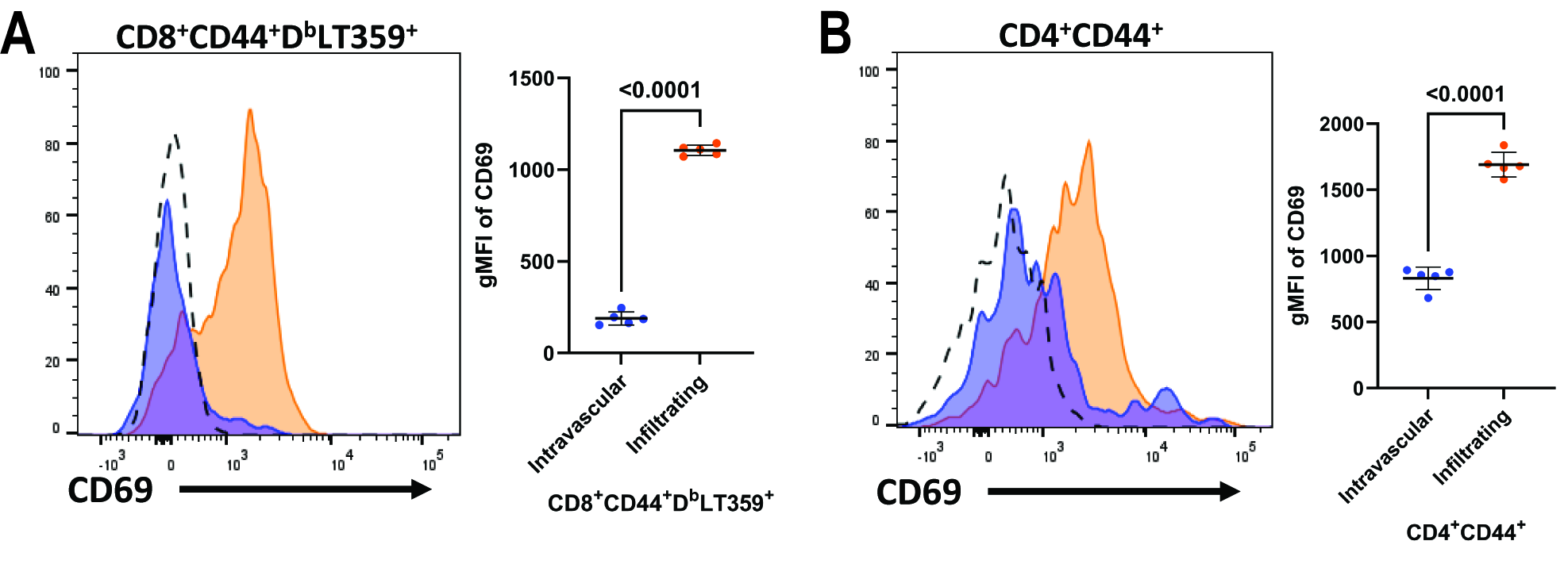

Supplement: S1 Fig — (A) Expression of CD69 on intravascular and infiltrating CD8+ CD44+ Db-LT359 tetramer+ T cells 30 dpi. Data are representative of three independent experiments (n = 5). (B) Expression of CD69 on intravascular and infiltrating CD4+ CD44+ T cells 30 dpi. Data are representative of three independent experiments (n = 5). Data are from two independent experiments (n = 10). Data were analyzed by paired t tests. (TIF) [file ppat.1012969.s001.tif]

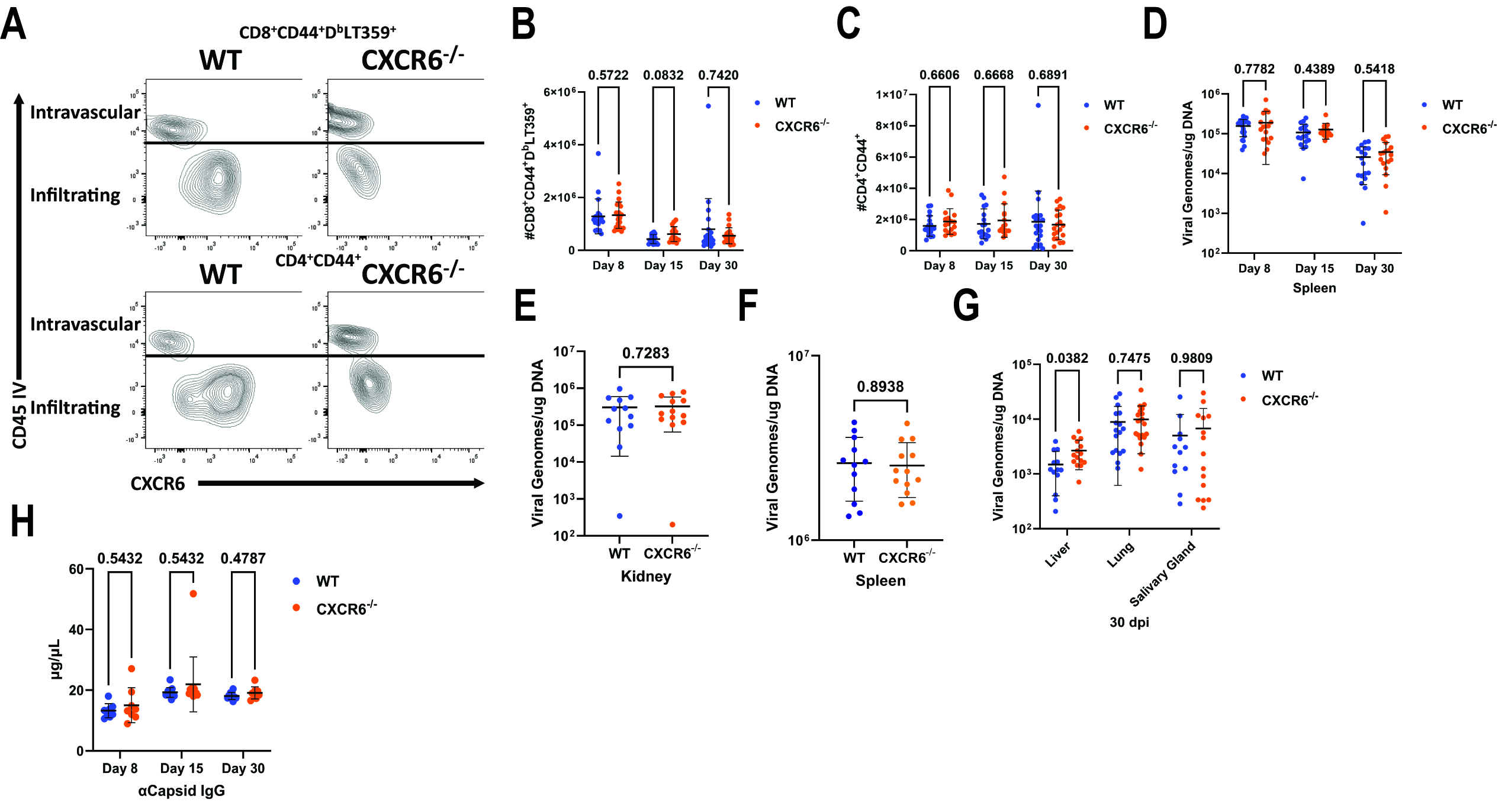

Supplement: S2 Fig — (A) Comparison of CXCR6 expression on intravascular and infiltrating kidney CD8+ and CD4+ WT and CXCR6−/− T cells. (B) Number of CD8+ CD44+ Db-LT359 tetramer+ T cells in the spleens of WT and CXCR6−/− mice 8, 15, and 30 dpi. Data are from 4-5 independent experiments (n = 16-21). (C) Number of CD4+ CD44+ T cells in the spleens of WT and CXCR6−/− mice 8, 15, and 30 dpi. Data are from 4-5 independent experiments (n = 16-21). (D) Virus levels in the spleens of WT and CXCR6−/− mice 8, 15, and 30 dpi. Data are from four independent experiments (n = 16-19). (E) Virus levels in the kidney of WT and CXCR6−/− mice 4 dpi. Data are from three independent experiments (n = 12-13). (F) Virus levels in the spleen of WT and CXCR6−/− mice 4 dpi. Data are from three independent experiments (n = 12-13). (G) Virus levels in the liver, lung, and salivary gland of WT and CXCR6−/− mice 8, 15, and 30 dpi. Data are from 3-4 independent experiments (n = 12-20). (H) ELISA for anti-capsid IgG in the serum of WT and CXCR6−/− mice 8, 15, and 30 dpi. Data are from 2-3 independent experiments (n = 8-13). Data were analyzed by multiple Mann-Whitney tests (B-D, G), Mann-Whitney tests (E-F), and multiple t test (H). (TIF) [file ppat.1012969.s002.tif]

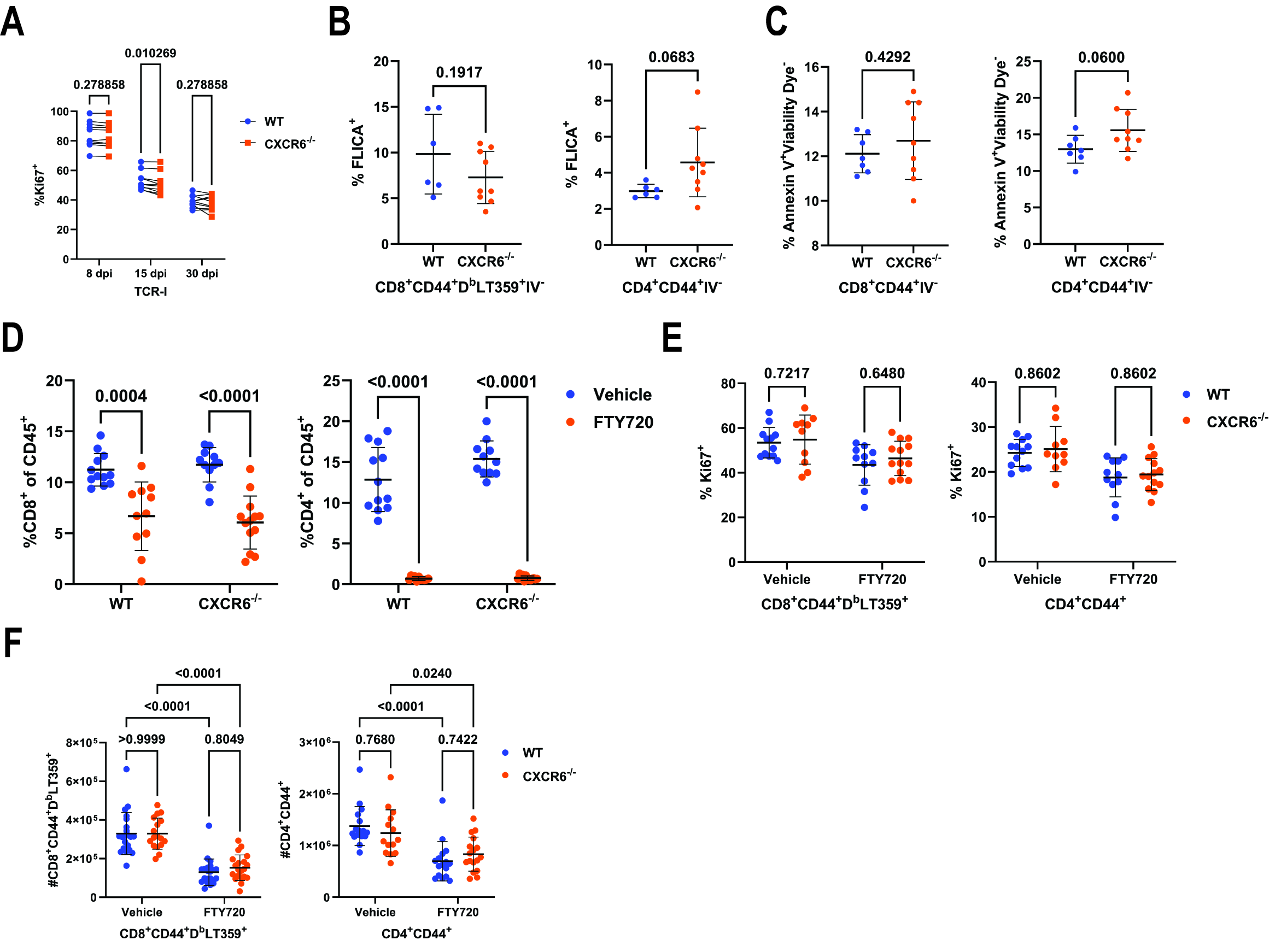

Supplement: S3 Fig — (A) Frequency of Ki67+ WT and CXCR6−/− TCR-I cells in the spleen of recipients 8, 15, and 30 dpi. Data are from two independent experiments (n = 10). (B) Kidney-infiltrating CD8+ CD44+ Db-LT359 tetramer+ and CD4+ CD44+ T cells stained with FLICA to identify activated caspase 3/7. Data are from two independent experiments (n = 6-9). (C) Kidney CD8+ and CD4+ T cells stained with viability dye and Annexin V to identify apoptotic (Annexin V+ viability dye-) cells in WT and CXCR6−/− mice 8 dpi. Data are from two independent experiments (n = 7-9). (D) Frequency of CD8+ and CD4+ cells out of total CD45+ cells in the blood of WT and CXCR6−/− mice treated with vehicle or FTY720. Data are from three independent experiments (n = 10-13). (E) Frequency of splenic CD8+ CD44+ Db-LT359 tetramer+ and CD4+ CD44+ T cells that are Ki67+ in WT and CXCR6−/− mice with vehicle or FTY720 treatment. Data are from three independent experiments (n = 10-13). Data were analyzed by multiple paired t tests (A), unpaired t tests (B-C), or multiple unpaired t tests (D-E). (TIF) [file ppat.1012969.s003.tif]

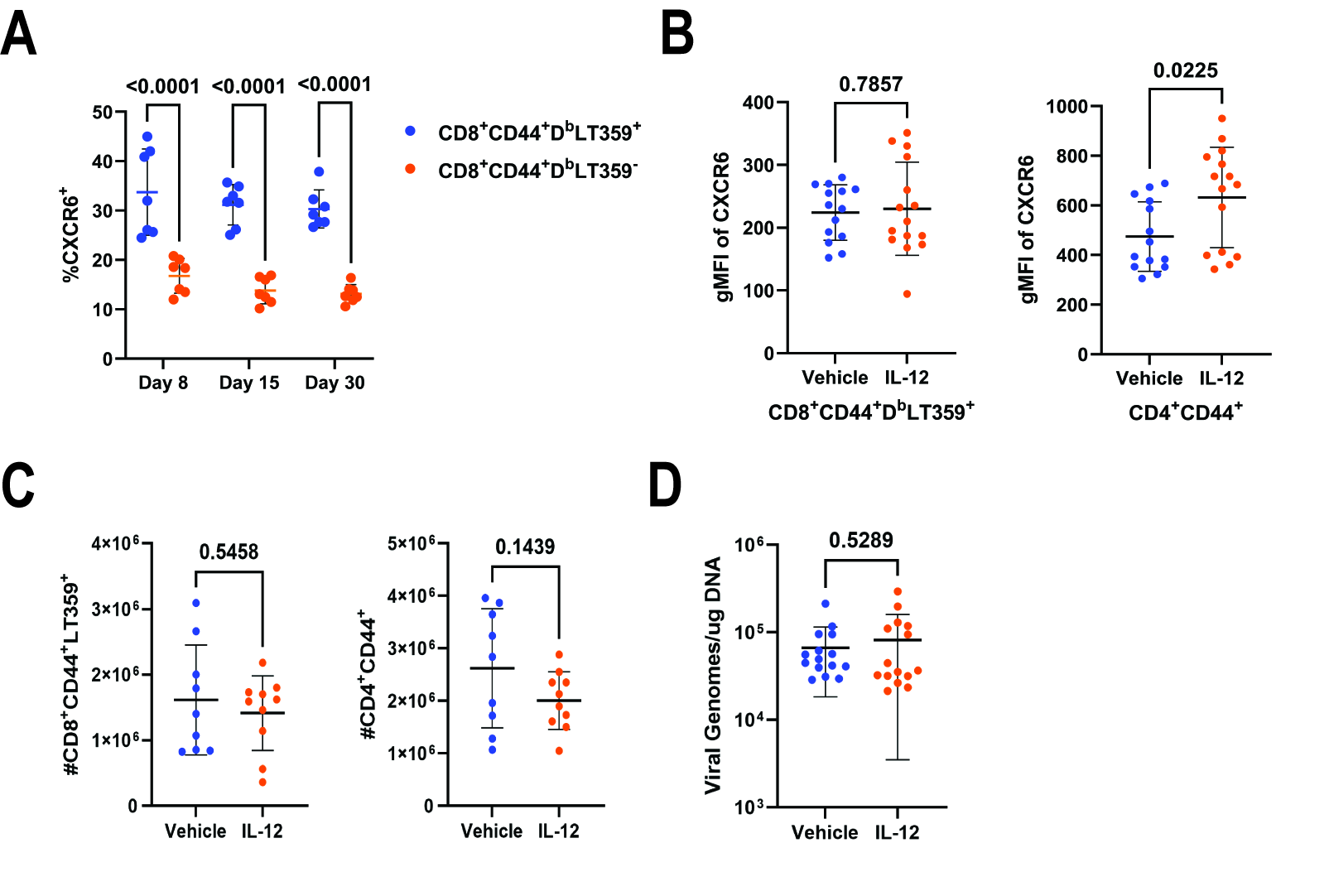

Supplement: S4 Fig — (A) Frequency of CXCR6 expression on Db-LT359 tetramer+ and Db-LT359 tetramer- CD8+ CD44+ T cells in the spleens of WT mice 8, 15, and 30 dpi. Data are from two independent experiments (n = 7). (B) CXCR6 expression on spleen CD8+CD44+ Db-LT359 tetramer+ T cells and CD4+CD44+ T cells after treatment with PBS vehicle or IL-12. Data are from three independent experiments (n = 14-15). (C) CD8+ CD44+ Db-LT359 tetramer+ T cell and CD4+ CD44+ T cell numbers after treatment with vehicle or IL-12. Data are from two independent experiments (n = 9). (D) Virus DNA genomes in spleen after vehicle or IL-12 treatment. Data are from three independent experiments (n = 14-15). Data were analyzed by two-way ANOVA (A) or unpaired t test (B-D). (TIF) [file ppat.1012969.s004.tif]
