# Supplementary material for: The CXCR6-CXCL16 axis mediates T cell control of polyomavirus infection in the kidney
Source: PLoS Pathog. 2025 Mar 5;21(3):e1012969. doi: 10.1371/journal.ppat.1012969 (PMC11922244; doi:10.1371/journal.ppat.1012969)
Supplement: Attachment — Submitted filename: Response to Reviewers.docx [file ppat.1012969.s006.docx]

We thank the reviewers for their positive comments on our manuscript and appreciate their thoughtful suggestions to further amplify the clinical implications of this work. As detailed below, we have revised our manuscript to address each of the points raised by the three reviewers.

Reviewer 1:

1. Model for why MuPyV and LCMV trafficking are specific to the kidney? Why the kidney, is it special/different compared to other organs? If not, how many other organs/tissues have specific chemokine code for homing T cells?

We, too, are intrigued by the involvement of particular chemokine-chemokine receptor axes in the migration of T cells to specific organs. In the Introduction, **(lines 75-82)** we described a variety of these axes that are involved in trafficking of T cells to different tissues. We have added to the Discussion a paragraph **(lines 293-304)** describing a model for involvement of the CXCR6-CXCL16 axis to the kidney based on induction of metalloproteases by MuPyV-infected cells to release of the soluble form of CXCL16. We speculate that such a mechanism may also apply to other pathogens that use this chemokine receptor-chemokine axis.

2. Explain rationale for composition of panel of “variety” of chemokine receptors that were included. Difficult to discern if they were cherry picked or not.

We would like to assure the reviewer that interrogating the intra- and extra-vascular distribution of nine chemokine receptors was a far more comprehensive panel than used in prior publications by others. We selected this panel based on the literature of chemokine receptors expressed by T cells that guide their infiltration of tissues infected by different microbial pathogens. We have added a sentence describing our rationale for picking this panel in the Results **(lines 106-109).**

3. Increased soluble form not evident in all 5 mice, only 3 at best. Can you rule out loading variation, normalized quantification would be helpful. Include a clearer image at minimum.

We apologize for our oversight in not stating that loading controls were included. The increase observed in the soluble form of CXCL16 was not due to variations in loading, as the β-actin loading control was evenly expressed. The quantification of the band intensity in Figure 2 was calculated by normalizing the expression of soluble CXCL16 against the β-actin loading control. We have added a sentence describing this to the Results **(lines 136-137),** to the Figure Legend **(lines 663-665**) and to the Materials and Methods **(lines 412-413).**

Reviewer 2:

1. The human data is not convincing as presented in terms of the role of the CXCL16:CCR6 signaling axis in CD8+ T cell recruitment to the kidneys.

We moved Supplemental Table 1 to Table 1 in the main text to provide easier access for the reader to the data used to generate the graph in Figure 6D. We also clarified in the Results detailing that the values in Figure 6D were calculated by comparing kidney transplant patients with PVAN to those with stable graft function (**lines 264-267**).

2. If the authors could discuss in more detail how targeting this chemokine signaling pathway could be used for ameliorating disease in humans. It would increase the potential clinical importance of the findings provided.

We believe that these results have valuable clinical relevance for adoptive cellular immunotherapy for kidney allograft recipients with PVAN. As recommended, we have expanded on this point in the Discussion (**lines 344-349**) by citing a recent paper demonstrating the beneficial effect of elevating CXCR6 in antigen-specific T cells for adoptive immunotherapy for pancreatic cancer. We also speculated that systemic anti-IL-12 may be an intervention to check trafficking of CXCR6^+^ anti-donor T cells in patients with PVAN (**lines 342-343**). In the Discussion we had also described how the *ex vivo* administration of IL-12 could increase the expression of CXCR6 on adoptively transferred virus-specific T cells to control PVAN (**lines 334-336**).

Reviewer 3:

1. The class II tetramer data demonstrating CXCR6 expression on virus-specific CD4 T cells in the kidney is important and should be moved from the supplement figures to figure 1.

We agree and have moved the CD4 T cell tetramer staining in the kidney from Supplemental Figure 1 to Figure 1 in the main text. We added a Fig 1C and revised the numbers of the other panels in this figure and added text to the legend for Fig 1 (lines **648-651**).

2. In figure 3e, are the comparisons between WT and CXCR6 KO cells made comparing total TCR-I T cells, or only cells with a TRM phenotype (CD69+ CD103+)? If made with total T cells, is there a similar defect observed in CXCR6 KO cells when looking at only cells with a TRM phenotype?

Text has been added to the Figure Legend of Figure 3E to clarify the groups being compared (**line 681**). The comparisons between WT and CXCR6 KO TCR-I cells are made between total TCR-I cells and not markers for a resident memory phenotype.

3. The graph in figure 6d is difficult to interpret. Are all these data points significant, and what are the comparisons being made? The summary in supplemental table 1 is more clear, could these data be included in figure 6?

Please see our response to a similar comment from Reviewer 2. We have revised Figure 6D and its legend (**lines 727-729**) to explicitly indicate the statistical significance of each comparison using asterisks, with further details in Table 1 (formerly Supplemental Table 1). Non-significant comparisons are those where the 95% confidence interval of the log-fold change includes 0. All the comparisons are made for expression of *CXCL16* and *CXCR6* in kidney transplant (KT) biopsies from patients with PVAN and stable graft function.
